# A Case of Coombs-Negative Primary Warm Autoimmune Hemolytic Anemia Due to IgA Antibody That Responded Well to Rituximab and Steroids

**DOI:** 10.7759/cureus.63598

**Published:** 2024-07-01

**Authors:** Sanjay Rao Gergal Gopalkrishna Rao

**Affiliations:** 1 Internal Medicine, State University of New York (SUNY) Upstate Medical University, Syracuse, USA

**Keywords:** autoimmune hemolysis, iga hemolytic anemia, extended coombs test, autoimmune hemolytic anemia (aiha), coombs' negative

## Abstract

Warm autoimmune hemolytic anemia (WAIHA) occurs due to antibodies active at body temperature that react with antigens on the surface of red blood cells, leading to hemolysis. Antibodies are typically IgG. WAIHA, associated exclusively with IgA antibodies, remains rare. Direct antiglobulin (Coombs) test may result negative in IgA antibody associated WAIHA. IgA-mediated WAIHA can present with severe hemolytic anemia. Further testing using an expanded direct antiglobulin test (DAT) panel is necessary to detect IgA antibodies if there is a high suspicion of autoimmune hemolytic anemia in cases that initially test negative for DAT. Steroids with or without rituximab are the mainstay of treatment. Early detection using an extended DAT panel with monospecific antisera helps avoid further investigations, unnecessary transfusions, and complications.

## Introduction

Autoimmune hemolytic anemia (AIHA) is an acquired clinical condition caused by autoantibodies that react with antigens on the surface of red blood cells (RBCs), leading to hemolysis. The most common type of AIHA is warm AIHA (WAIHA), which is caused by antibodies that are effective at body temperature and constitutes approximately 70-80% of cases [1,2]. The autoantibodies can arise spontaneously or in the setting of a condition or medication and are typically IgG [2]. Up to 14% of patients with WAIHA can have IgA autoantibodies, but these are commonly detected alongside IgG or IgM [3,4]. WAIHA, associated exclusively with IgA antibodies, remains rare [3]. A positive direct antiglobulin test (DAT) in a person with hemolytic anemia is confirmatory of WAIHA. WAIHA with negative DAT is rare and is believed to occur in less than 5% of people with WAIHA [5-7]. Individuals with suspected DAT-negative WAIHA, who present with new-onset hemolytic anemia without any other evident cause, need to undergo further testing with an extended DAT panel to detect antibodies of the IgA or IgM isotype as these antibodies may be missed by the routine DAT. IgA-mediated WAIHA can present with severe hemolytic anemia and a negative DAT [8]. Steroids with or without rituximab are the mainstay of treatment [4,7]. Early detection using an extended DAT panel with monospecific antisera helps in avoiding further investigations and provides efficient management.

We present a case of DAT-negative primary WAIHA due to IgA antibody that responded well to rituximab and steroids.

## Case presentation

A 61-year-old male was admitted due to shortness of breath with exertion and dark urine. The symptoms appeared gradually over a period of two weeks and got progressively worse. He denied any nausea, vomiting, abdominal pain, diarrhea, changes in stool color, bloody sputum or emesis, skin rash, or petechiae. There was no recent travel, new drug exposure, pertinent environmental exposure, chemical contact, or significant family history.

Hemoglobin (Hb) on admission was 8.5 g/dL (baseline 10-11 g/dL). Total bilirubin was 4.8 mg/dL, and direct bilirubin was 0.2 mg/dL. Lactate dehydrogenase (LDH) was elevated at 983 U/L. Reticulocyte percentage was 20.1% and absolute reticulocyte was 520.3x10^3^/uL. Prothrombin time (PT) and activated partial thromboplastin time (aPTT) were within normal limits (13.8 and 26.4 seconds, respectively).

Hematology service was consulted for further workup. An extensive workup was done to evaluate genetic, infectious, and immunogenic causes of hemolytic anemia. Iron panel was normal with iron saturation 29%, ferritin 400 ng/mL, serum iron 82 ug/dL, total iron-binding capacity 294 ug/dL, and serum transferrin 212 mg/dL. Folate and B12 were 15.8 ng/mL and 334 pg/mL, respectively, indicating no deficiency. Direct Coombs test was negative. Malaria smear and G6PD were also negative. Peripheral smear showed spherocytosis with polychromasia, as well as nucleated RBCs without schistocytes or Heinz bodies. Cardiolipin and glycoprotein antibodies were negative. Antinuclear antibody (ANA) was positive at 1:160. Haptoglobin was low at 15 mg/dL. Hex phase was mildly elevated at 8.5 seconds. Flow cytometry showed no evidence of acute leukemia or non-Hodkin’s lymphoma. Kappa and lambda were mildly elevated, but the ratio was normal at 1.36. Parvovirus IgG was positive but IgM was negative. Cytomegalovirus and Epstein-Barr virus were negative. Copper level was normal at 129 ug/dL (Table 1). Osmotic fragility test showed fragile RBCs with evidence of hemolysis. Abdominal ultrasound showed mild splenic enlargement. Comprehensive hematology panel to check for RBC enzymes was sent out to Mayo Clinic. CT angiography (CTA) of the chest showed a small filling defect in the bilateral lower lobes, suggestive of pulmonary embolism, and the patient was started on heparin drip and later transitioned to rivaroxaban. Echocardiogram showed no valvular abnormalities. Complete blood count (CBC), haptoglobin, and LDH were serially monitored. He was started on folic acid. Once Hb was stable, he was discharged with outpatient hematology follow-up.

**Table 1:** Laboratory results with reference ranges

Lab results	
Hemoglobin (Hb)	8.5 g/dL (11.5-15.5 g/dL)
Total Bilirubin	4.8 mg/dL (<1.2 mg/dL)
Direct bilirubin	0.2 mg/dL (<0.3 mg/dL)
Lactate dehydrogenase (LDH)	983 U/L (112-214 U/L)
Reticulocyte percentage	20.1% (0.6-2.8%)
Absolute reticulocyte	520.3x10^3^/uL (26-122x10^3^/uL)
Prothrombin time	13.8 sec (11.6-14 sec)
Activated partial thromboplastin time	26.4 sec (23.3-31.7 sec)
Iron saturation	29% (20-55%)
Ferritin	400 ng/mL (30-400 ng/mL)
Serum iron	82 ug/dL (59-158 ug/dL)
Total iron-binding capacity	294 ug/dL (278-500 ug/dL)
Serum transferrin	212 mg/dL (200-360 mg/dL)
Folate	15.8 ng/mL (>4.77 ng/mL)
B12	334 pg/mL (211-946 pg/mL)
Haptoglobin	15 mg/dL (30-200 mg/dL)
Hex phase	8.5 sec (<8 sec)
Kappa:lambda ratio	1.36. (0.26-1.65)
Copper level	129 ug/dL (69-132 ug/dL)

Labs were monitored regularly on an outpatient basis: CBC, comprehensive metabolic panel (CMP), LDH, and reticulocytes. He required intermittent blood transfusions of around 1-2 units pRBCs (packed RBCs) each month. Comprehensive hematology panel that was sent out to Mayo Clinic showed normal RBC enzyme levels. Hb electrophoresis was normal with HbA 97.1%, HbF 0.5%, and HbA2 2.4%. He continued to have low-grade hemolysis with improvement in Hb. Genetic workup was sent. Labs were monitored regularly.

He was hospitalized again after a few months for cholecystitis and underwent laparoscopic cholecystectomy. During this episode, his Hb had been around 6-7 g/dL. He was not responding appropriately to pRBC transfusions. He had indirect hyperbilirubinemia (4.6 mg/dL), elevated LDH (944 U/L), low haptoglobin (<15 mg/dL), and negative Coombs test. His rivaroxaban was held on admission for cholecystectomy, but he was found to have a new recurrent pulmonary embolism. Rivaroxaban was restarted, and a decision was made for lifelong anticoagulation.

Invitae anemia hemolytic panel showed ABCG8 heterozygous variant. Since this mutation is associated with Sitosterolemia, and this can cause hemolytic anemia and macrothrombocytopenia, Mayo Clinic panel for sterols was sent. Mayo Clinic sterol panel came back as normal. Also, his cholesterol levels were normal, thus ruling out sitosterolemia. Hb was around 7 g/dL and CMP showed continued indirect hyperbilirubinemia (4.3 mg/dL). Donath Landsteiner Antibody testing was conducted and was negative. He was started on prednisone 60 mg/d. His Hb improved to 9 g/dL. After around six weeks, prednisone was tapered to 50 mg daily with 10 mg taper every week until 20 mg and then by 5 mg every week. Steroids were tapered off. However, there was a return of hemolytic anemia (Hb 7-8.5 g/dL).

Extended Coombs test to check for IgA WAIHA was then sent to the blood bank of Wisconsin, which came back positive. He was started on rituximab weekly for four doses and prednisone 60 mg/d with, plan until Hb >10 g/dL and then gradual taper over two to three months. Hb has now been steadily improving and consistently >10 g/dL over several weeks (Table 2).

**Table 2:** Hemoglobin during different phases

Hemoglobin results	
Prior to treatment	7- 8.5 mg/dL
With steroids	9- 10.5 mg/dL
With discontinuation of steroids	7- 8.5 mg/dL
With rituximab and steroids	>10 mg/dL

## Discussion

Our case demonstrates the importance of performing additional testing with an extended DAT panel to identify antibodies of the IgA isotype if there is a strong suspicion of AIHA in apparently DAT-negative cases. The DAT involves washing the patient's RBCs and then exposing them to antiserum or monoclonal antibodies directed against immunoglobulins, specifically IgG and a portion of the complement C3d. The cause of a negative DAT in WAIHA could be due to (i) IgG molecules bound to RBCs at a level that is too low to be detected by the DAT (ii) IgG autoantibodies with low affinity that are removed from the red cells when they are washed during the testing process and (iii) IgA autoantibodies bound to RBCs and occasional warm IgM autoantibodies that are not detected by the standard anti-human globulin reagent. [9]. In the absence of positive DAT, once other causes of hemolysis are ruled out using pertinent investigations, additional testing with an extended DAT panel is necessary to detect the existence of IgA autoantibodies bound to the surface of RBCs to diagnose IgA-mediated WAIHA. WAIHA, mediated by IgA, may manifest as severe hemolytic anemia with a negative DAT, and the primary treatment involves steroids, with or without rituximab [4,7,8]. In this case, the oral steroid therapy resulted in a positive outcome for our patient. However, his anemia returned with discontinuation of steroids. His clinical condition improved with rituximab and steroids, eliminating the need for additional packed RBC transfusions. Additionally, there was an increase in Hb concentration and a decrease in reticulocyte counts and serum lactate dehydrogenase levels. In conclusion, early diagnosis of DAT-negative, IgA-mediated WAIHA with the extended DAT panel can help avoid further tests, unnecessary transfusions, and complications.

## Conclusions

IgA-mediated WAIHA can present as a diagnostic challenge due to the negative DAT test. If the DAT results are negative, and there is a strong clinical suspicion of WAIHA, it is necessary to conduct further testing with an extended DAT panel using monospecific antisera to detect antibodies of the IgA type. Early diagnosis of IgA-mediated WAIHA can help avoid further tests, unnecessary transfusions, and complications.
